# An Amyloidogenic Fragment of the Spike Protein from SARS-CoV‑2 Virus Stimulates the Aggregation and Toxicity of Parkinson’s Disease Protein Alpha-Synuclein

**DOI:** 10.1021/acschemneuro.5c00478

**Published:** 2025-08-22

**Authors:** João Flavio Gemignani, Paulo Augusto Netz, Daniel Izecksohn, David Dabkiewicz, Ming-Hao Li, Adalgisa Felippe Wiecikowski, David Eliezer, Yraima Cordeiro, Cristian Follmer

**Affiliations:** † Laboratory of Biological Chemistry of Neurodegenerative Disorders, Department of Physical Chemistry, Institute of Chemistry, 28125 Federal University of Rio de Janeiro, Rio de Janeiro 21941-909, Brazil; ‡ Institute of Chemistry, Federal University of Rio Grande do Sul, Porto Alegre 91501-970, Brazil; § Department of Biochemistry, 12295 Weill Cornell Medical College, New York, New York 10065, United States; ∥ Faculty of Pharmacy, Federal University of Rio de Janeiro, Rio de Janeiro 21941-902, Brazil

**Keywords:** alpha-synuclein, amyloid-beta, spike, SARS-CoV-2, Parkinson’s disease, Alzheimer’s disease

## Abstract

Emerging evidence suggests that the severe acute respiratory syndrome coronavirus 2 (SARS-CoV-2) infection may have long-term deleterious effects on the central nervous system and even contribute to post-COVID neurological syndromes. Interestingly, inflammation-induced proteolytic processing of the Spike protein of SARS-CoV-2 leads to the generation of peptides capable of aggregating into amyloid fibrils *in vitro*. Herein, we investigate the *in vitro* effect of a fibrillogenic fragment of the Spike protein [Spike 194–203 (S194)] on the aggregation and toxicity of the Parkinson’s disease (PD) protein α-synuclein (αSyn). Our results indicate that S194 fibrils stimulate in a concentration-dependent manner the fibrillation of αSyn monomer, resulting in aggregates with increased capacity of inducing lipid vesicle leakage and toxicity to neuroblastoma cells, in comparison with either αSyn or S194 alone. Bidimensional NMR (^1^H–^15^N-HSQC) suggests that S194 fibrils cause a higher perturbation in both the N-terminal region (sequence: 19–68) and the hydrophobic central domain of the αSyn monomer (sequence: 71–95), which is corroborated by protein–peptide docking and molecular dynamics simulations. In contrast with fibrils from wild-type αSyn, aggregates from the PD variant A30P exhibited a remarkable accelerative effect on S194 fibrillation. Similarly, fibrils from amyloid-β peptides, which are linked to Alzheimer’s disease, exhibited a pro-aggregating effect on the S194 monomer. Taken together, these findings might contribute to a broader understanding of the potential connections between SARS-CoV-2 infection and amyloid-related neurodegenerative disorders, highlighting areas that may warrant further investigation.

## Graphical abstract

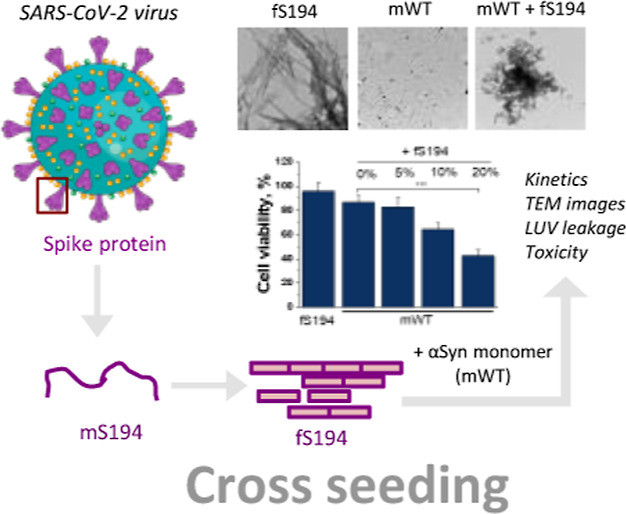

## Introduction

The severe acute respiratory syndrome coronavirus 2 (SARS-CoV-2) caused a global pandemic of coronavirus disease in 2019 (COVID-19), resulting in millions of infections and deaths worldwide. While the primary symptoms of COVID-19 are related to the respiratory tract, there is growing concern about the long-term neurological consequences of SARS-CoV-2 infection, including cognitive deficits, olfactory dysfunction, fatigue, and even the development or aggravation of neurodegenerative diseases.^1,2^ In this scenario, SARS-CoV-2 was shown to infect both human brain organoids and brains of mice expressing human angiotensin converting enzyme 2 (ACE-2),^3^ the receptor for the virus in the cell. Importantly, alterations in cerebral microstructure linked with post-COVID condition have been identified in patients.^4^ For instance, neuroinflammation and neuronal injury were associated with the infection of neurons in the central nervous system (CNS) by SARS-CoV-2.^3,4^ These findings raise important questions about the neurologic sequelae of SARS-CoV-2 infection, which are reinforced by recent data suggesting that SARS-CoV-2 infection could increase the risk for neurodegenerative diseases.^5^

Post-COVID-19 parkinsonism or the aggravation of motor symptoms of Parkinson’s disease (PD) patients was identified after SARS-CoV-2 infection.^6^ A connection between viral infection and PD was first described following the influenza A pandemic of 1918, which was associated with an outbreak of postencephalitic parkinsonism.^7^ The infection by other viruses including hepatitis C, HIV, and West Nile virus was also described to increase the potential of developing neurological disorders via mechanisms that have been not yet fully understood.^8,9^

Symptoms of PD are caused by the degeneration of dopaminergic neurons in the substantia nigra pars compacta (SNpc), leading to a reduction in dopamine levels and the characteristic motor impairment.^10^ The histological hallmark of PD is the presence of intracellular inclusions composed by fibrillar aggregates of the protein α-synuclein (αSyn), denoted as Lewy bodies (LBs).^11^ αSyn is a disordered monomeric protein (14 kDa) found predominantly in the presynaptic membrane of nerve cells and plays a role in the transport, fusion, and release of synaptic vesicles.^12^ Under native conditions, the protein behaves predominantly as an intrinsically disordered monomer, which can undergo fibrillation due to genetic mutations, overexpression, specific posttranslational modifications, or interaction with other proteins.^13^

The ability of amyloid fibrils to propagate and amplify the aggregation of other proteins underscores the complexity of protein misfolding and the interconnected nature of neurodegenerative disorders. For instance, *in vitro* studies showed that αSyn fibrils can initiate the aggregation of amyloid-β (Aβ) peptides by providing a surface for the recruitment and assembly of monomeric peptides into potentially toxic oligomers and fibrils. On the other hand, fibrils from Aβ can induce the fibrillation αSyn *in vitro*.^14^ The formation of extracellular plaques composed by fibrils of Aβ is one of the main pathological features of Alzheimer’s disease (AD). In this scenario, a hypothetical connection between COVID-19 and PD may be associated with the interaction of proteins from the virus with αSyn. Certain proteins from SARS-CoV-2, such as the S1 receptor-binding domain (S1 RBD) and the nucleocapsid protein (N-protein), were found to interact with αSyn and promote its fibrillation.^15,16^ Interestingly, the spike protein of the SARS-CoV-2 virus forms fibrillogenic fragments *in vitro* upon its proteolytic digestion by action of the enzyme neutrophil elastase (NE),^17^ which is linked to the host inflammatory response. One of these fragments is Spike194-203 (S194) (1244 Da) (sequence: FKNIDGYFKI), which is unique for SARS-CoV-2. Nevertheless, the effect of the amyloid fibrils of S194 on the aggregation of αSyn remains undetermined.

Herein, we evaluate the effect of S194 fibrils (fS194) on the aggregation of the wild-type (WT) αSyn monomer (mWT). Our findings indicate that fS194 stimulates in a concentration-dependent manner the aggregation of mWT, forming structures capable of causing lipid vesicle leakage and with increased toxicity to neuroblastoma cells in comparison with the proteins alone. In addition, NMR experiments and computational simulations indicate that the interaction of fS194 occurs preferentially with the N-terminal and central hydrophobic domains of the αSyn monomer. We also evaluated the kinetics of fibrillation of the familial mutants of αSyn, A30P and A53T (related to early onset PD) and amyloid-β peptides when they are coincubated with S194. Overall, our findings suggest that S194 might act synergistically with other amyloidogenic proteins to promote the acceleration of fibrillation processes, which may be relevant for understating the molecular basis of neurodegenerative events associated with SARS-CoV-2 infection.

## Results

### Fibrils of S194 Stimulate the Aggregation of aSyn

Aggregates of S194 exhibit enhanced signal of Thioflavin-T (ThT) fluorescence, one important characteristic of amyloid fibrils, despite the absence of a sigmoidal curve for kinetics of aggregation of the protein, as shown in the Supporting Information (Figure S1). Curiously, after S194 fibrillation, no significant alteration of the secondary structure of the protein was detected by using far-UV circular dichroism (CD) (data not shown), which may result from sample heterogeneity, phase separation, light scattering effects, and other variables that impede the reliable quantification of its secondary structure by CD. The ability of fS194 to stimulate the aggregation of mWT was investigated, as schematized in Figure 1, upper scheme. The expression of the concentration of fS194 in the mixture in molarity unit gives a misinterpretation because S194 is in aggregated form, while αSyn is monomeric. For this reason, we utilized the concentration of the fibrils in relation to the monomers in terms of percentage of total protein mass. We notice that fS194 caused an increase in the relative ThT fluorescence signal in a concentration-dependent manner when it was coincubated with mWT (Figure 1A). No significant variation in the ThT signal was observed for mWT incubated alone for until 50 h. The analysis of the kinetics parameters indicates that the maximum rate of the fibrillation of mWT rises from 0.028 h^–1^ to 0.045 h^–1^ when the percentage of fS194 increases from 5 to 20%. In addition, it was followed by a 3-fold decay of the duration of lag time (Figure 1B and C). It is worth noting that, in contrast to the results described above, the incubation of mS194 with mWT had no observable effect on the fibrillation process (data not shown). The analysis of the morphology of the aggregates by using transmission electron microscopy (TEM) indicated the formation of typical fibrillar aggregates for fS194, whereas highly fragmented/branched aggregates were observed for fS194 plus mWT (Figure 1D). No extensive protein aggregation was noticed for the incubation of mWT alone. On the other hand, coincubation of mS194 with fibrils of WT αSyn (fWT) did not produce any accelerative effect on the aggregation of the spike fragment (Figures S1). In addition, TEM images indicate that fWT displays a large fibrillar structure, while no significant changes were noticed for fWT plus mS194.

**Figure 1:**
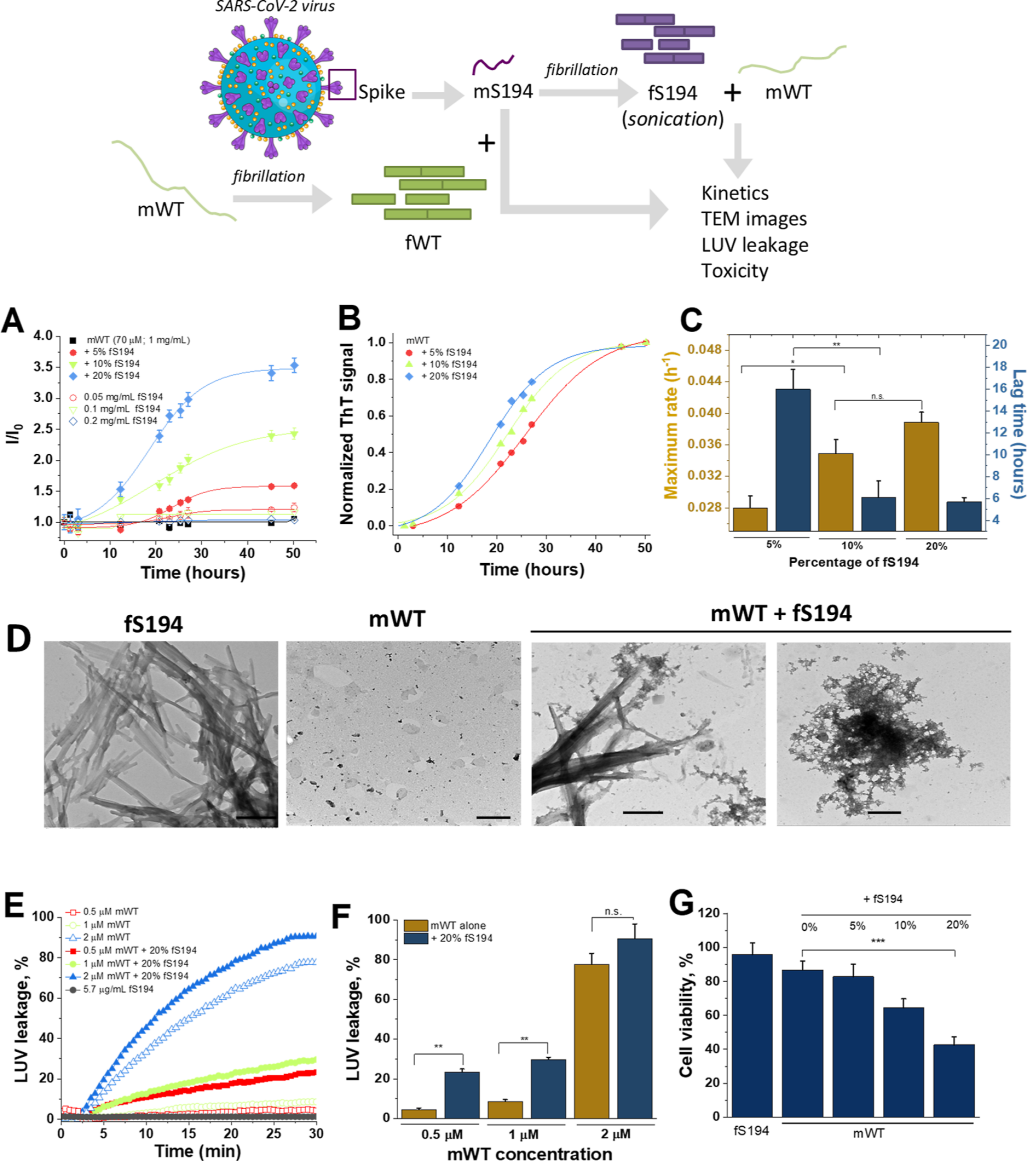
Fibrils of S194 stimulate the aggregation of the αSyn-WT monomer. Upper panel shows the scheme of the experimental design for the evaluation of the effect fS194 on the fibrillation of mWT. (A) Relative intensity of the ThT signal for the incubation of 70 μM of mWT in the absence or the presence of 5, 10, or 20% (m/m) of fS194 at 37 °C under agitation (300 rpm). (B) Normalized ThT curves and (C) kinetics parameters (maximum rate and lag time duration) for the aggregation of mWT in the presence of fS194 obtained in A. (D) TEM images of aggregates at the end of the incubation time (scale bars: 500 nm). (E) The effect of the concentration of either mWT or mWT plus 20% of fS194 (incubated for 24 h) on the leakage of LUVs. A concentration of fS194 of 5.7 μg/mL corresponds to the higher concentration of these species tested in the presence of mWT, i.e., 20% of fS194. (F) Percentage of the leakage of LUVs in the steady state caused by either mWT alone or mWT plus 20% of fS194. (G) Toxicity to neuroblastoma cells (SH-SY5Y) of mWT (20 μM) incubated during 24 h in the absence or the presence of 5, 10, or 20% of fS194. Results in A and C are mean ± standard deviation (SD) of six independent experiments, while in F and G are expressed as mean ± SD of three experiments. **p <0.01; 0.01< *p < 0.05; ns: not significant. Virus image in the upper panel was created by using Servier Medical Art templates, which are licensed under a Creative Commons Attribution 3.0 Unported License; https://smart.servier.com.

Alpha-syn interacts with lipid bilayers, altering their structure and dynamics, which may lead to increased membrane permeability. It means that the protein, particularly in certain oligomeric states, plays a crucial role in the integrity and function of cellular lipid membranes. In this context, we evaluate the effect of either mWT or mWT plus 20% of fS194 on the leakage of large unilamellar vesicles (LUVs) composed of 100% DOPG. Unlike fS194 alone, mWT alone was capable of inducing concentration dependently an increase in LUV permeability. On the other hand, aggregates generated by the coincubation of mWT with 20% of fS194 exhibited a higher effect on membrane permeability than mWT alone, notably at lowest concentrations (0.5 and 1 μM of mWT plus 20% of fS194) (Figure 1E and F). For instance, while 0.5 and 1 μM mWT promote 5% and 8% leakage, respectively, mWT plus 20% of fS194 caused a leakage of 22 and 25%. For the higher concentration of mWT tested here (2 μM), there was no statistical difference between the samples incubated with or without 20% of fS194.

Next, the toxicity to neuroblastoma cells (SH-SY5Y) of mWT incubated during 24 h in the absence or the presence of varying concentrations of fS194 was investigated (Figure 1G). None of the effect on cell viability was observed in the treatment with either 0.05 mg/mL of fS194 (equivalent to 45 μM of mS194) or 20 μM of mWT alone. However, an increase in the toxicity was observed for mWT preincubated with increasing concentrations of fS194. For instance, cell viability decreased from 82 to 40% when fS194 increased from 5 to 20% of the total protein. Collectively, these data indicate the aggregates generated upon the coincubation of mWT with fS194 exhibit a higher capacity of both stimulating the permeability of LUV and reducing the viability of neuroblastoma cells, in comparison with either mWT or fS194 alone.

### Fibrils of S194 Interact with N-Terminal and Central Hydrophobic Domains of αSyn

To get information about the residues of the αSyn monomer involved in the interaction with fS194, we applied ^1^H–^15^N-HSQC spectroscopy. Figures 2A and B show the superposition of spectra of ^15^N-mWT alone versus ^15^N-mWT plus fS194 at the inital time and after 48 h of incubation, respectively. In comparison with the initial time (Figure 2A), we observed a remarkable loss of intensity after 48 h of the coincubation of mWT plus fS194 relative to mWT alone. Two regions were preferentially affected by the presence of fS194: amino acid sequences 19–68 and 71–95 (Figure 2C). The sequence 71–77 (VTGVTAV), the most affected by fS194, belongs to the nonamyloid-β component (NAC) domain (residues 61–95) of αSyn, which is rich in nonpolar residues, particularly Val residues, making it highly hydrophobic [as indicated by the hydropathicity index (Figure 2D)] and essential for the protein aggregation. These data suggest that the interaction of fS194 with the αSyn monomer occurs preferentially into the hydrophobic domain of the protein in a manner that facilitates its fibrillation.

**Figure 2:**
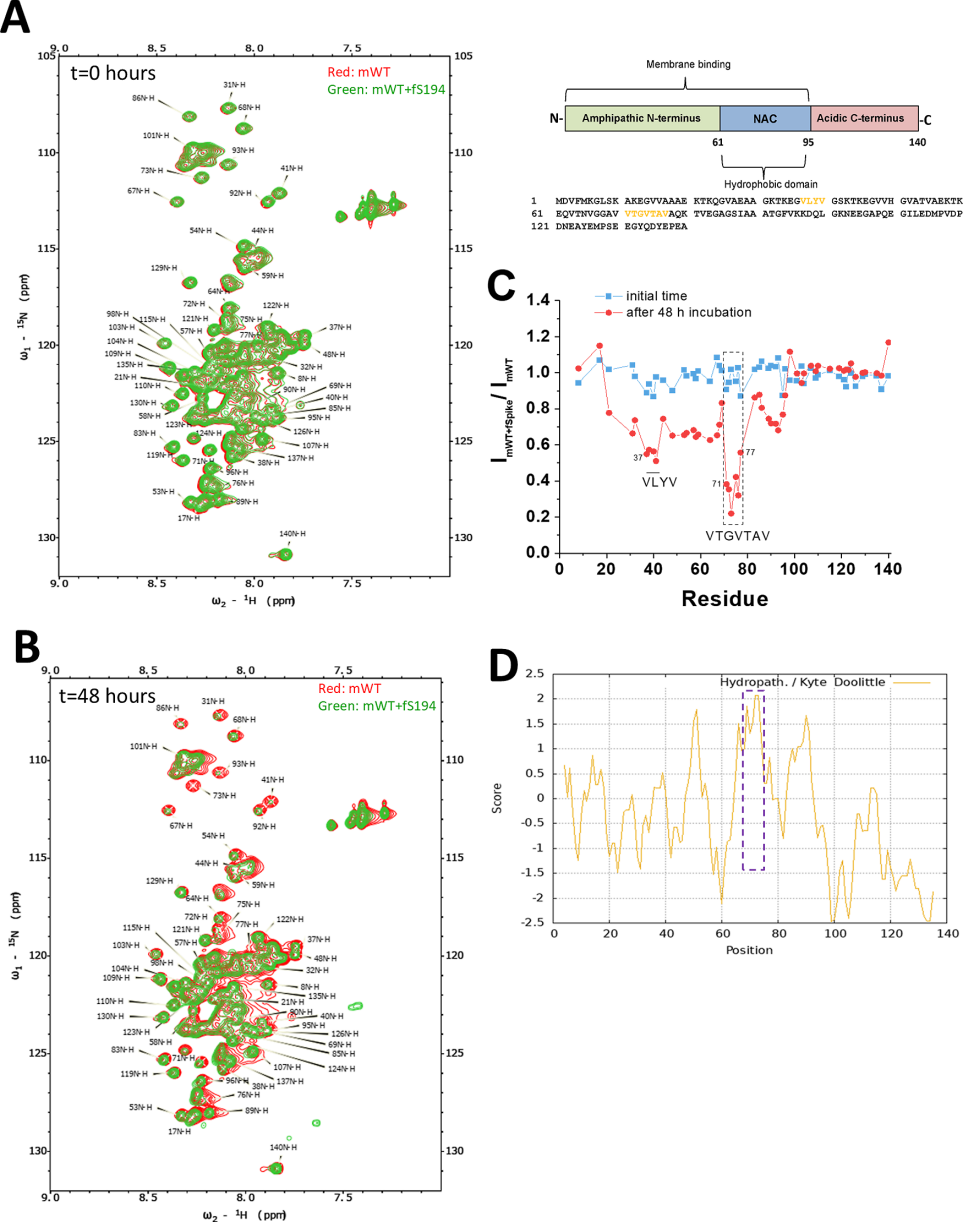
^1^H–^15^N-HSQC analyses of mWT in the presence of fS194. Spectra of 200 μM of mWT (red), in 10 mM NaPB, pH 7.4, 0.14 M NaCl, 2.7 mM KCl, 10% D_2_O, superposed with mWT plus fS194 (green) (equimolar concentrations), acquired in the initial time (A) or after 48 h of incubation at 37 °C and agitation (B). (C) Ratio of peak intensities of (mWT + fS194)/mWT at the initial time and after 48 h incubation. The upper panel shows αSyn domains and the protein sequence, in which the most affected residues by fS194 are shown in dark yellow. (D) Hydropathy index of the αSyn sequence (https://web.expasy.org/protscale/). Spectra were recorded at 10 °C.

To provide additional insights into the interaction between αSyn and S194 monomers, we utilized molecular docking calculations using AutoDock Vina. Either the theoretical or experimental model for the fibrils of S194 is not available to be utilized as the initial structure for the computational studies. For this reason, we decided to evaluate how the interaction between the two proteins in monomeric forms occurs and compare these results with those findings obtained using NMR, in which the fibrils of S194 were incubated with monomeric αSyn. Either starting from experimental (micelle-bound αSyn) or simulated αSyn monomer structures as a receptor (Figure 3A and 3B, respectively), the results pointed out to a preferential interaction of the S194 peptide with the hydrophobic, valine-rich portions of the αSyn unfolded monomer [from 3000 ns molecular dynamics (MD) simulation] starting in residue 37 (VLYV) or in residue 71 (VTGVTAV). The most relevant interacting αSyn amino acid residues were GLU20, VAL26, GLU28, THR33, LYS34, VAL37, TYR39, GLY41, LYS43, LYS45, HIS50, VAL71, THR72, GLY73, VAL74, GLN79, ALA91, PHE94, LYS97, and MET116. When the micelle-bound αSyn structure was used, the interaction with S194 occurred only near the first site (37–40). Starting from the docked structures (using equilibrated αSyn as a receptor), two series, each of them with three replicates, with a total of six 250 ns long classical MD simulations, were carried out. The results confirm the stability of the interaction between αSyn and S194, since for most of the systems, the root-mean-square deviation (RMSD) showed low values and tendency to structural equilibrium (Figure 3C), and the distance between the ligand and protein was essentially constant along the simulation (Figure 3D). The results of the remaining replicates are shown in the Supporting Information (Figures S2–S4) and essentially support this picture, with the S194 peptide showing only temporary migrations away from αSyn. Moreover, a substantial number of hydrogen bonds between αSyn and S194 was steadily present, as can be seen in Figure 3E. The strength of the interaction was quantified by the free energy of binding along the MD trajectory, calculated using the MM/PBSA method. The average free energy of binding considering all the starting structures and replicates was −30.2 kcal/mol, implying a strong interaction. This method also allows a heatmap-like residue decomposition analysis (Figure 3F), which shows that the most relevant αSyn residues for the interaction with S194 were VAL26, VAL37, VAL74, THR92, GLY93, and PHE94. Residue decomposition analysis for the other replicates is shown in the Supporting Information (Figures S5 and S6). There is a variability in the relevant interacting residues among the simulations, nevertheless the most prevalent interacting residue types in αSyn are valine and lysine. Considering that MM/PBSA lacks a reliable approach for the estimate of the entropy, the strength of interaction may be overestimated. Therefore, the binding free energy was also calculated as a function of an order parameter (in this case, the minimum distance between αSyn and S194), following the approach of Bellaiche and Best^18^ (see details in the Material and Methods). The calculated binding free energy was, respectively, 22.6 and 23.9 kJ/mol for model 1 and model 2 as an average over all replicates. The structural stability of the αSyn was also quantified by calculating the radius of gyration (Rg) and solvent-accessible surface area (SASA). Table 1 shows the detailed results (Number of H-Bonds, Free Energy of Binding, Radius of Gyration, and Solvent Accessible Surface Area) for all six simulations. Our simulation results, concerning Rg and SASA, are like those obtained by Chesney et al.^19^

**Figure 3:**
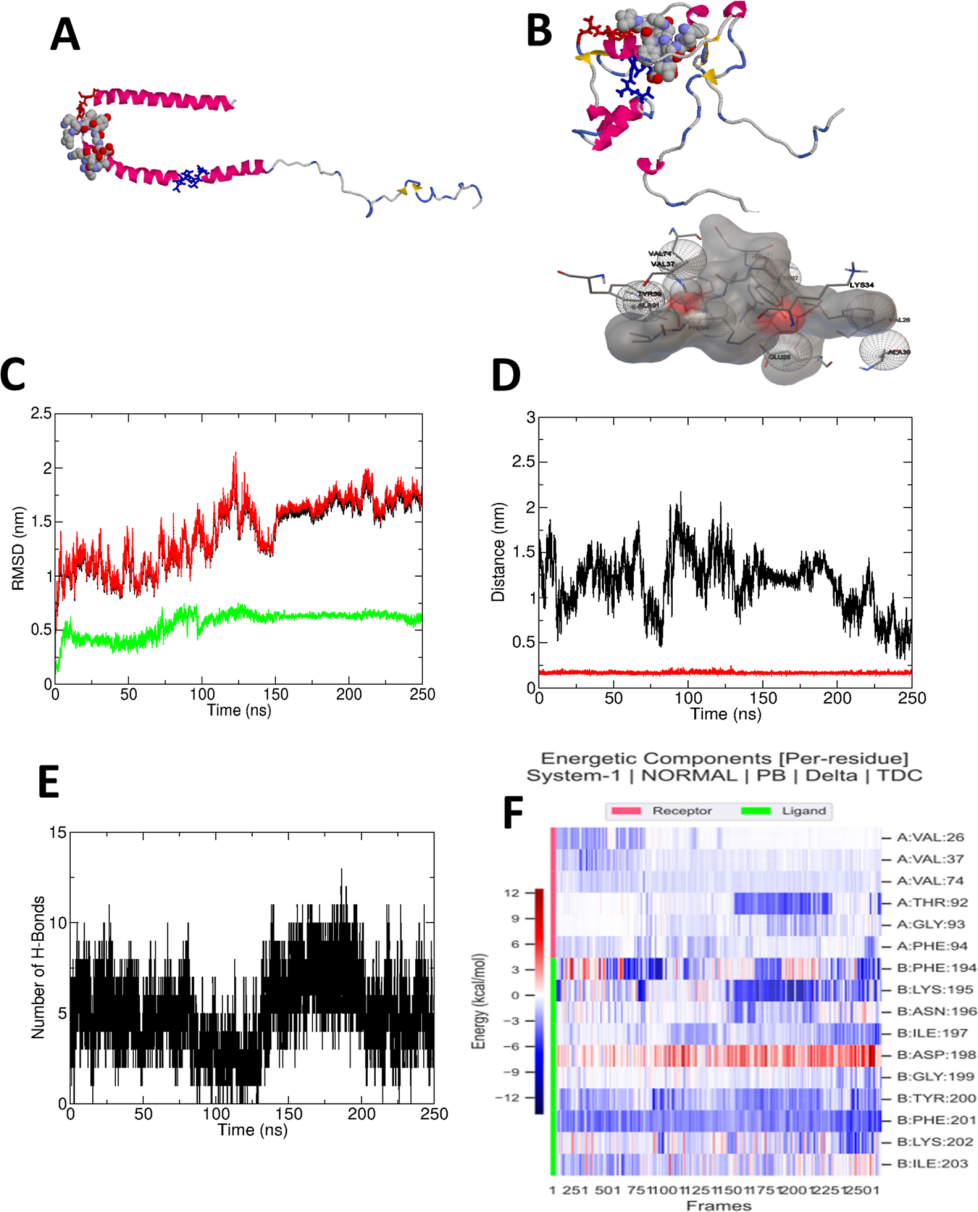
Docking and molecular dynamics. (A) Docking results: structure of αSyn PDB-ID 1XQ8 shown as cartoon. Residues V37LYV are shown in wireframe red and V71TGVTAV are shown in wireframe blue. Best ranked docked S194 configuration generated with AutoDock Vina shown as spacefill. (B) Same as before, for an optimized receptor structure, obtained as one among the end structures of extensive molecular dynamics simulations. Below, details of the pattern of interacting residues, generated with AutoDock Tools. (C) Root mean square deviation (RMSD) along the MD trajectory. Black: whole complex, red: αSyn, green: S194. (D) Center of mass distance (black) and minimum distance (red) between the ligand (S194) and receptor (αSyn). (E) Number of hydrogen bonds along the molecular dynamics trajectory. (F) Free-energy decomposition analysis showing the most relevant residues with favorable (blue) or unfavorable (red) interactions.

**Table 1:** Parameters of the Interaction (Number of H-Bonds, Free Energy of Binding, Radius of Gyration, and Solvent Accessible Surface Area) between αSyn and mS194 Obtained from Six Simulations

system		H-bonds	Δ*G*_binding_ (kcal mol^–1^)	radius of gyration (nm)	solvent-accessible surface area (nm^2^)
model 1	replica 1	3.3 ± 2.5	–9.75 ± 15.8	2.2 ± 0.3	119.8 ± 5.6
	replica 2	6.1 ± 2.5	–42.0 ± 15.0	1.9 ± 0.1	123.3 ± 5.2
	replica 3	3.1 ± 2.0	–25.9 ± 10.7	2.0 ± 0.2	121.2 ± 7.3
model 2	replica 1	5.0 ± 2.0	–36.5 ± 11.9	2.3 ± 0.3	123.1 ± 6.0
	replica 2	5.7 ± 2.5	–39.5 ± 13.1	2.0 ± 0.3	118.6 ± 6.0
	replica 3	3.2 ± 2.6	–27.3 ± 14.1	2.8 ± 0.4	129.1 ± 6.5

It is worth noting that all of our simulations were performed using monomeric S194 instead of the corresponding fibrils, mainly because the structure of fS194 is not yet available. This means that the simplification in the S194 model should have a huge impact on the interaction with αSyn. Additionally, it is worth mentioning that we did not observe any significant effect on the kinetics of fibrillation when mS194 and mWT were coincubated. However, our molecular modeling data showed good agreement with the data obtained by NMR experiments, in which fS194 was used. Furthermore, the fact that S194 is a very short peptide (only 10 residues) leads us to speculate that most of the amino acids present in mS194 are also found on the surface of fS194. Therefore, whether the interaction of fS194 with αSyn depends on either some structural motif or just a key amino acid sequence in the peptide, regardless of whether it is present in the fibrils or in the S194 monomer, is still an open question.

### Fibrils of A30P Stimulate the Aggregation and Toxicity of S194

We next compared the effect of fS194 on the aggregation of WT versus the A30P and A53T mutants of αSyn. It was reported that mutations A30P and A53T exhibit distinct aggregation propensities and toxicities compared to the WT protein. While the A30P variant shows a decreased tendency to aggregate, forming fewer fibrils and more stable oligomers, the A53T significantly increases the aggregation propensity, leading to the formation of toxic oligomers and fibrils.^20^ For this reason, it is expected that these variants of αSyn might have distinct aggregating behavior when coincubated in the presence of S194. Figure 4A shows that the presence of 20% of fS194 promoted a slight accelerative effect on the aggregation of WT or A30P monomers without affecting the aggregation of mA53T, likely due to the high propensity of this variant to undergo fibrillation. We next investigated the kinetics of fibrillation when fibrils from WT, A30P, or A53T were incubated with mS194 (Figure 4B). While fA53T and fWT caused a slight inhibition in the fibrillation process of mS194 in comparison with mS194 alone, fA30P produced a remarkable fibrillogenic effect on mS194, in which the maximum rate of the fibrillation of mS194 increased proportionally with the fA30P concentration (Figure 4C). Interestingly, mS194 incubated with 20% of fA30P displayed a higher toxicity to neuroblastoma cells in comparison with either the proteins administrated separately or mS194 preincubated with fWT (Figure 4D). The analysis of the morphology of the fibrils by TEM revealed that fA30P plus mS194 displays thin fibrils, clearly distinct from that observed for fibrils of this mutant incubated alone in which the presence of small and branched fibrils mixed with amorphous aggregates was detected (Figure 4E). On the other hand, images from mS194 incubated with either fWT or fA53 revealed only subtle alteration of the morphology of the aggregates. The aggregates of fS194 plus mA53T exhibited morphology quite similarly to mA53T alone, consistent with the slight increase of ThT fluorescence observed for this mutant.

**Figure 4:**
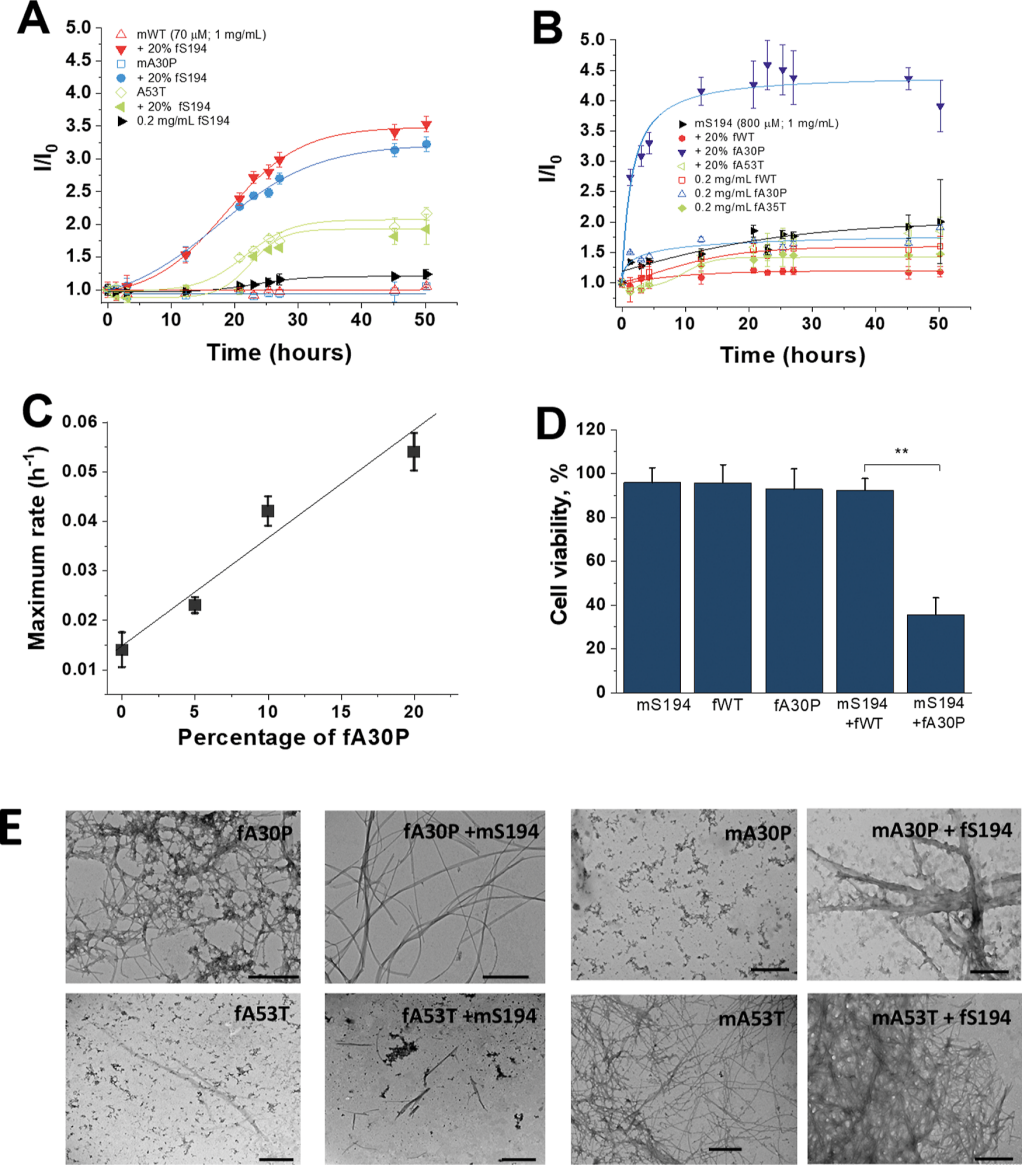
Effect of the coincubation of WT or the variants A30P or A53T of αSyn with S194. (A) Relative ThT signal during the incubation of 70 μM of monomeric αSyn (WT or variants) with or without 20% fS194 at 37 °C under agitation (300 rpm). (B) Kinetics of fibrillation of mS194 (800 μM) incubated with or without 20% of fWT, fA30P, or fA53T. (C) Effect of the percentage of fA30P on the maximum rate for the aggregation of mS194. Results in A and B are mean values ± SD from six independent experiments. (D) Evaluation of toxicity to neuroblastoma cells (SH-SY5Y) of mS194 (50 μM; 62.5 μg/mL) incubated for 24 h in the absence or the presence of 20% of fWT or fA30P (12.5 μg/mL or 0.875 μM of the monomer). Results in A–C are mean ± SD of six independent experiments, while in D are expressed as mean ± SD of three experiments. Symbols in D: **p <0.01; 0.01< *p < 0.05; ns: not significant. (E) TEM images of protein aggregates at the end of the incubation time (scale bars: 500 nm).

Amyloid fibrils are recognized for their high resistance to various proteases, and the examination of their proteolytic fragmentation is a common method for studying the structure of these aggregates.^21^ For this purpose, samples of monomeric αSyn incubated with 20% of fS194 were treated with either trypsin or proteinase K, and protein fragments were analyzed by using denaturing gel electrophoresis (SDS-PAGE). The proteolytic fragmentation of these samples was then compared with that obtained using typical αSyn fibrils (WT or mutants) (Figure S7). Although these experiments were done using the same αSyn concentrations (relative to the monomeric form), we cannot guarantee that the same quantity of fibrils is present. Therefore, our study only focused on analyzing the change in the relative intensity of the bands in the gel. Before the treatment with trypsin, two bands corresponding to αSyn and S194, both in a monomeric state, were visualized in SDS-PAGE (Figure S7, top). When compared with usual fWT, fibrils generated from mWT plus fS194 exhibited distinct cleavage peptides or peptides with different relative intensities upon incubation with either trypsin or proteinase-K. Trypsin-treated fA30P sample exhibited the same fragmentation profile as mA30P plus fS194. However, in the presence of proteinase-K, different protein fragments were generated from these samples, in which mA30P plus fS194 seemed to be totally digested, while some fragments were observed for fA30P. Fibrils obtained from the incubation of mA53T plus fS194 exhibited bands in the same position as fA53T, with only a minor change in the relative intensity of the bands generated from trypsin or proteinase-K digestion. We did not visualize any protein band in trypsin-treated fS194, probably due to the very small size of the peptide fragments. These findings suggest that the stimulating effect of seeds of fS194 on the aggregation of the αSyn monomer produces only subtle changes in the structure of the fibrils compared with typical fibrils of αSyn, which seems to be dependent on the αSyn variant.

We have used isothermal titration calorimetry (ITC) to confirm the existence of distinct interactions of mS194 with fWT versus fA30P, which could explain the quite different effects observed in Figure 4B. The heat release curves for mS194 titrated into a calorimeter cell containing fWT are shown in Figure S8. The negative heat change upon mS194 injection shows that the mS194–fWT interaction is exothermic with saturable sites and a change in enthalpy (Δ*H*) in the range of -200 to -150 kcal/mol. Interestingly, we did not observe any change in the Δ*H* when this experiment was done with fA30P. These data point out different thermodynamic contributions behind the interaction of mS194 with either fWT or fA30P. While the negative enthalpy of interaction seems to favor the binding of fWT to mS194, we can speculate that fA30P–mS194 interaction is likely driven by a gain in entropy (since enthalpy is not favorable), which is a characteristic of hydrophobic-driven interaction.

To gain further information on the remarkable effect of fA30P in comparison with fWT on the fibrillation of mS194, we analyzed the hydrodynamic radius (Rh) of the species that are found in the soluble fraction by using dynamic light scattering (DLS). Table 2 indicates that at the initial time (0 h of incubation), the fWT sample exhibits a wide distribution of sizes with Rh values of 12.8 nm (9.5%), 39 nm (35.6%), and 315 nm (54.8%), whereas the most abundant fraction of oligomers in the fA30P sample has 47.5 nm, which corresponds to ∼85% of the species for this variant. As expected, mS194 alone exhibited very small sizes with an Rh of 0.6 and 2.5 nm (52.3 and 47.7%, respectively). For fA30P plus mS194, more than 80% of species have small Rh sizes (20 to 80 nm), while this population drops to half in the case of fWT plus mS194. The presence of an enhanced population of small oligomers in fA30P fraction could explain why aggregates of this variant exhibited a high capacity of seeding mS194 aggregation. By analyzing the size distribution of the soluble species after a short incubation time (5 h), we noticed that mS194 plus fA30P presents a major population of species with small sizes (67% with 62 nm), while for mS194 plus fWT, the particles in this size range correspond only to 42%. These data reinforce the idea that small species are likely to work as more efficient seeds during the aggregation of mS194 compared to the large aggregates. Interestingly, mS194 incubated alone displayed only very large oligomers with Rh values of 792 and 6799 nm. Therefore, an increased population of small soluble species observed for fA30P incubated with mS194 could play an important role in the kinetics of fibrillation.

**Table 2:** DLS Analysis of the Species Present in the Soluble Fraction of fWT or fA30P Samples, Incubated or Not with mS194 at the Initial Time or after 5 h of Incubation at 37 °C and Agitation^a^

	0 h			5 h		
	Rh (nm)	% PDI	% intensity	Rh (nm)	% PDI	% intensity
*fWT*	12.8	3.6	9,5	0.5	2.7	0.4
	39.0	4.4	35.6	31.8	3.0	30.4
	315.2	2.8	54.8	122.8	3.3	69.2
*fWT* + *mS194*	0.6	0.9	0.2	1.0	0	1.1
	13.7	1.9	7.6	9.1	4.6	3.1
	35.7	4.5	40	73.5	4.1	42.3
	195.7	2.5	47	316.1	3.2	53.5
	1030.4	0.0	15.5			
*mS194*	0.6	3.9	52.3	15.1	0.3	1.3
	2.5	4.6	47.7	792.2	3.2	44.3
				6799.0	4.6	54.4
*fA30P*	3.1	2.5	0.5	1.1	2.6	0.2
	47.5	4.6	84.9	12.1	4.5	5.5
	374.3	1.2	16.6	44.5	5.1	49.1
				256.1	3.3	45.2
*fA30P* + *mS194*	0.9	4.4	0.3	0.6	4.5	9.7
	24.5	4.1	27.9	62.1	4.5	67.6
	76.9	3.9	55.2	305.8	3.8	22.7
	912.7	4.2	16.6			

a %PDI = polydispersity index.

### Fibrils of the Aβ Peptide Promote the Aggregation of mS194

The formation of extracellular plaques of the Aβ peptide is one of main pathological features of AD, in which deposits of the peptide can directly damage neurons and disrupt their function leading to cognitive decline and memory loss.^22^ However, the role of viral infection in the development of AD is not as clearly defined. Herein, we addressed the ability of fibrils from two Aβ peptides (Aβ_1–42_ and Aβ_25–35_) to stimulate the aggregation of mS194. Aβ_25–35_ is a shortened, synthetic fragment of Alzheimer’s Aβ peptide. This specific fragment is not typically found physiologically in the brain, but it is important in research studies to help researchers understand the mechanisms underlying the pathophysiology of AD. Figures 5A and B show that fibrils from either Aβ_1–42_ or Aβ_25–35_ peptides (fAβ_1–42_ and fAβ_25–35_, respectively) can promote an acceleration of mS194 aggregation. For the coincubation of mS194 with fAβ_1–42_, we noticed an increase of 2-fold ThT fluorescence intensity in comparison with the fibrillation of mS194 alone. In the case of mS194 with fAβ25–35, the ThT signal was 4-fold higher than the control. Negative-staining TEM revealed that fAβ_1–42_ formed tiny and short fibrillar aggregates, as well as a minor population of amorphous aggregates, contrasting with the fAβ_25–35_ peptide that formed highly heterogeneous branched aggregates (Figure 5C). Interestingly, when mS194 was incubated in the presence of fAβ_1–42_, we visualize fibrils with a highly homogeneous morphology, mostly thin and elongated unbranched fibrils. For fAβ_25–35_ plus mS194, a dense network of short and branched fibrils was noticed, apparently less amorphous than that observed for fAβ_25–35_ alone. Afterward, we analyzed the effect of fS194 on the aggregation kinetics of mAβ peptides (Figure 5D and E). In this case, the aggregation of mAβ_1–42_ seems to be only marginally perturbed by the presence of fS194, while fS194 seems to negatively affect the aggregation of mAβ_25–35_ in comparison with mAβ_25–35_ incubated alone. Collectively, these findings suggest that mS194 is prone to fibrillate more rapidly when it is in the presence of fAβ, whereas fS194 appears not to affect mAβ fibrillation, in contrast to what was found for incubation of fS194 with the αSyn monomer.

**Figure 5:**
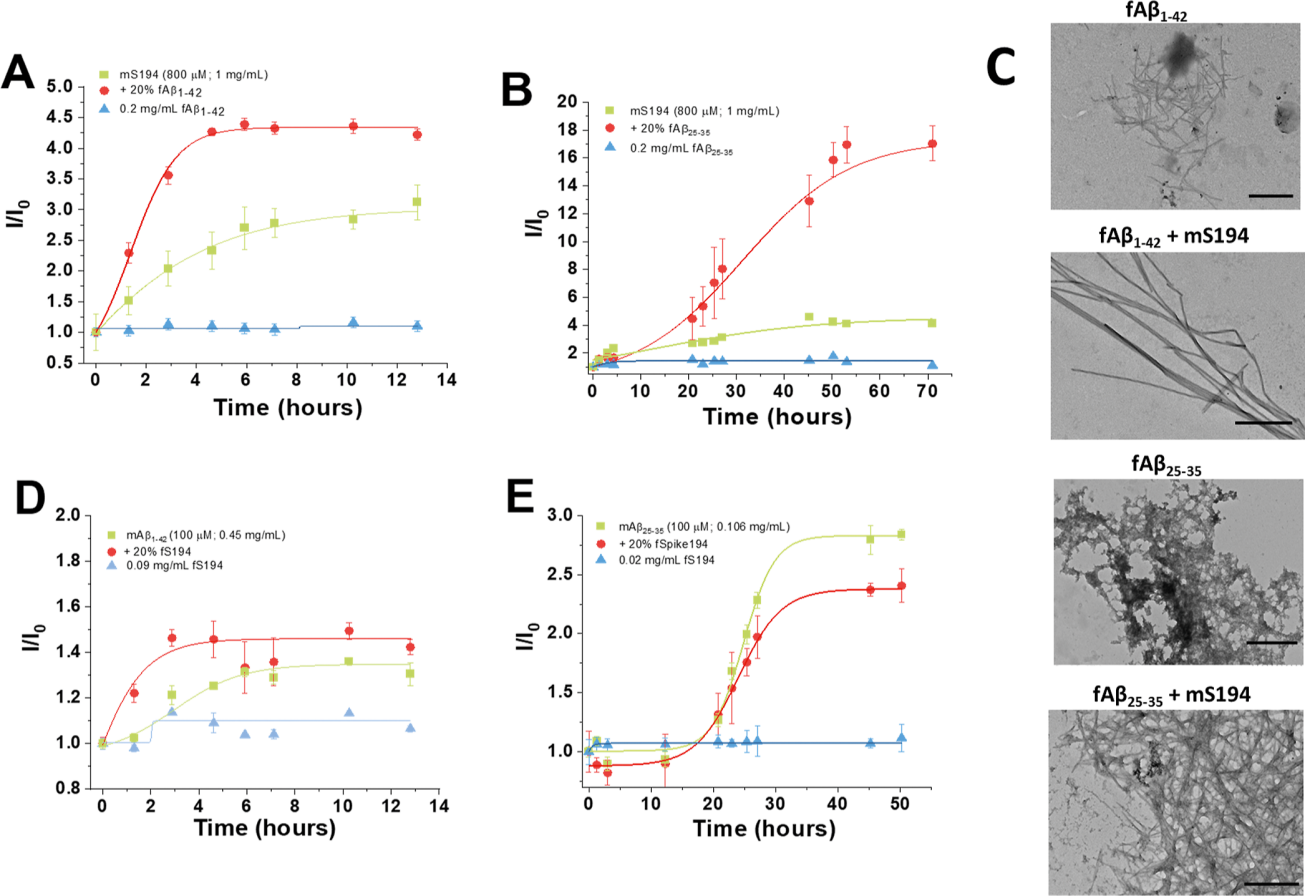
Crosstalk between Aβ peptides and S194. Panels (A) and (B) show the aggregation of 800 μM of mS194 in the presence of 20% of fAβ_1–42_ or fAβ_25–35_, respectively. (C) TEM images of protein aggregates at the end of the incubation time (scale bars: 500 nm). Aggregation kinetics of 100 μM mAβ_1–42_ (D) or mAβ_25–35_ (E) incubated in the presence of 20% of fS194. Results of the kinetics curves are expressed as mean values ± SD from six independent experiments.

## Discussion

Several theoretical and experimental studies have suggested that certain proteins from the SARS-CoV-2 virus can promote, either directly or indirectly, the formation of amyloid aggregates. For instance, the S1 domain of SARS-CoV-2, which includes the fragment S194, has been shown to interact with αSyn, inducing the formation of protein aggregates capable of causing synaptic damage and cytotoxicity in a cellular model of synucleinopathy.^23^ Interestingly, S1 domain and αSyn aggregates colocalize in HEK293 cells.^23^ Similarly, the SARS-CoV-2 N-protein interacts with αSyn, accelerating its fibrillation.^16^ Various viral infections were linked to the induction of αSyn or Aβ aggregation. For example, H1N1 infection has been reported to induce αSyn aggregation and inhibit autophagy in dopaminergic neurons, thereby increasing susceptibility to neurodegeneration.^24^ *In vitro* and animal model studies have demonstrated that the spike protein of HSV-1 enhances aggregation of the Aβ_1–42_ peptide.^25^

Unlike the findings presented above, our study explores the cross-seeding phenomenon between SARS-CoV-2 protein fragments and human amyloidogenic proteins. Importantly, our results highlight a significant impact of coaggregation between fS194 and mWT on the toxicity of the resulting aggregates. Notably, in contrast with the interaction between fS194 and αSyn monomers, we found that fS194 does not affect the aggregation of Aβ_1–42_ or Aβ_25–35_, whereas fAβ fibrils can act as seeds for the aggregation of the S194 monomer. Conversely, other spike protein fragments appear to influence the Aβ aggregation. For example, *in vitro* and computational studies show that a short spike fragment containing the sequence _1058_HGVVFLHVTYV (S1058) enhances aggregation and toxicity of Aβ_1–42_.^26^ Collectively, these data indicate that there are no universal characteristics in terms of the fibrillogenic effect of the interaction of SARS-COV-2 proteins with amyloidogenic human proteins.

One critical question arising from our results concerns the physiological relevance of the concentrations of the spike fragment and αSyn used in our experiments. The concentration of αSyn in neurons, particularly within presynaptic terminals, is relatively high, potentially reaching up to 1% of the total cytosolic protein in the brain. While the exact physiological concentration of αSyn varies, it is estimated to be around 22–50 μM within the synapse.^27^ It is important to note that αSyn exists in a dynamic equilibrium between a soluble state and a membrane-bound form, leading to a variability in its local concentration within the cell. Regarding S194, we estimated that the maximum intracellular concentration of spike protein monomers could reach approximately 24.9 μM. This estimate reflects spike monomers incorporated into virions and underscores the high production capacity of infected host cells. However, the accumulation and localization of individual SARS-CoV-2 proteins within specific cellular compartments remain unclear. In our experiments, we coincubated 70 μM αSyn monomer with 5–20% (m/m) of fS194, which means that mS194 concentrations are from 40 to 160 μM. This concentration range produced the most pronounced cross-seeding effects, which is comparable to estimated physiological concentrations of these proteins (in terms of the concentration ratio of S194 per αSyn). In another experimental setup, αSyn fibrils (0.2 mg/mL or ∼14 μM relative to the monomeric form) were incubated with a high concentration of mS194 (800 μM). Although this concentration of mS194 is likely beyond physiological relevance, no change in the mS194 fibrillation rate was observed in the presence of fWT.

## Conclusions

Our results indicate that fS194 can act as seeds for the fibrillation of mWT, leading to aggregate species that cause lipid vesicle leakage and exhibit increased toxicity to neuroblastoma cells in comparison to the proteins alone. NMR experiments suggest that the interaction of fS194 occurs preferentially with the N-terminal and central hydrophobic domains of mWT. While fA53T and fWT caused a slight inhibition of the fibrillation of mS194, fA30P produced a concentration-dependent acceleration of the fibrillation of mS194. The increased abundance of small oligomeric species in the fA30P fraction may account for the strong ability of these aggregates to promote mS194 aggregation. In addition, mS194 incubated with fA30P displayed a higher toxicity to neuroblastoma cells in comparison with either the proteins administrated separately or mS194 preincubated with fWT. Finally, our results showed that mS194 is prone to fibrillate more rapidly when it is in the presence of fAβ_1–42_ or fAβ_25–35_, whereas fS194 appears not to affect mAβ fibrillation. Overall, our findings raise compelling questions about the potential role of SARS-CoV-2 infection in accelerating or triggering neurodegenerative diseases linked to protein amyloidosis. They underscore the urgent need for in vivo studies to uncover whether fragments of the Spike protein can drive pathological cross-seeding with αSyn and contribute to long-term neurological consequences.

## Materials and Methods

### Expression and Purification of Recombinant αSyn and Aβ_1–42_ Monomers

The expression and purification of αSyn were carried out as previously described by our group.^28^ The concentration of the protein was determined by measuring absorbance at 276 nm with a molar extinction coefficient of 5600 M^–1^ cm^–1^. To produce the Aβ_1–42_ peptide, the plasmid pET vector [pET-Sac-Abeta­(M1–42)] was transformed into BL21­(DE3)­pLysS (Thermo Fisher Scientific, USA) and the bacteria were grown at 37 °C, under agitation at 180 rpm and induced with 1 mM IPTG for 4 h. The peptide was purified based on a previously described procedure.^29^ The molar concentration of the Aβ_1–42_ monomer was determined by measuring absorbance at 276 nm with a molar extinction coefficient of 1460 M^–1^ cm^–1^.

### Production of Fibrils

Fibrils of αSyn were produced by incubation of the monomer at 200 μM in 10 mM sodium phosphate, pH 7.4, 2.7 mM KCl, 140 mM NaCl (PBS buffer) at 37 °C under agitation (450 rpm) in a 96-well microplate using thermomixer equipment (Eppendorf, Germany). The aggregation of Aβ_1–42_ was done in 20 mM HEPES, pH 6.5. To produce fAβ_25–35_, 1 mg of the synthetic peptide (GenScript, USA) was solubilized in 200 μL of trifluoroacetic acid, agitated during 1 h at 10 °C, and then dried in nitrogen gas, followed by 3 h under vacuum in a Savant SpeedVac vacuum concentrator (Thermo Fisher Scientific, USA). To generate fAβ_25–35_, 100 μM peptide was solubilized in 25 mM HEPES at pH 6.8 and kept at 25 °C, under agitation at 350 rpm for 3 days. Fibrils of S194 were produced by incubation of 1 mg of the synthetic peptide (GenScript, USA) in 50 μL of 100% hexafluoroisopropanol (HFIP) at 0 °C during 10 min and then solubilized in PBS buffer at a final protein concentration of 800 μM (1 mg/mL) with 5% HFIP. Fibrils of S194 were generated by agitating the solution (600 rpm) at 37 °C for 24 h. The formation of fibrils of all proteins was confirmed by mixing the protein solution with 5 μM ThT and measuring the probe fluorescence by excitation at 446 nm and measuring the emission at 485 nm using a Cary Eclipse Fluorimeter (Agilent Technologies, USA).

### Digestion of Fibrils with Proteinase-K and Trypsin

Protein samples were sonicated for 10 min and treated with 50 μg/mL of trypsin from porcine pancreas or 2 μg/mL of proteinase K (Merck, USA) at 37 °C during 30 min^21^ The reaction was stopped by addition of SDS sample buffer and then boiling for 5 min. The trypsin/proteinase K digestion profile was analyzed by 18% SDS-PAGE.

### Dynamic Light Scattering (DLS)

Samples were centrifuged at 10.000*g* at 25 °C for 10 min to remove insoluble aggregates. Soluble fractions were analyzed with a DynaPro NanoStar equipment (Wyatt Technology, USA) in a 45 μL-quartz cuvette. Three measurements with ten accumulations each were recorded for each sample. Hydrodynamic radii (Rh) and mass percentages of species were calculated from the autocorrelation curves with the software Dynamics 7.1.5 (Wyatt Technology, USA) by regularization analysis.

### NMR Experiments

^1^H–^15^N HSQC spectra were acquired using a Bruker AVANCE 500 MHz spectrometer (Weill Cornell NMR Core), equipped with a Bruker TCI cryoprobe, at a temperature of 10 °C. Spectra were recorded for two samples: one containing 200 μM mWT and another containing 200 μM mWT mixed with an equimolar concentration of fS194. After the initial spectra were collected, both samples were subjected to agitation at 37 °C and 350 rpm for 48 h, and HSQC spectra were recorded again. Data processing was performed using NMRpipe^30^ and spectra were analyzed with NMRFAM-Sparky.^31^ Backbone resonance assignments from previous studies were utilized for the analysis.^32^

### Isothermal Titration Calorimetry (ITC)

Direct interaction of mS194 with fWT or fA30P was investigated by ITC in an ITC-200 microcalorimeter (Malvern MicroCal, USA) at 25 °C. Two microliters of mS194 (at 200 μM in the syringe) were sequentially titrated into a 10 μM solution of fibrils of αSyn in the cell at an interval of 180 s. Stirring speed was set at 750 rpm. Both mS194 and αSyn samples were dissolved in PBS buffer with 5% HFIP. The amount of heat generated per titration was determined by integrating the area under the peaks. Heat of dilution of mS194 in buffer and of buffer titrated into αSyn in the cell was measured separately and the larger heat was subtracted from the titration data.

### Vesicle Leakage

Large unilamellar vesicles (LUVs) were prepared by an extrusion method^33^ by using two-syringe extruder equipped with a polycarbonate filter of 80 nm pore size. For this purpose, 10 mg of DOPG [1,2-di-(9Z-octadecenoyl)-*sn*-glycero-3-phospho-(1′rac-glycerol) (Avant Polar Lipids, USA)] was solubilized in 100 μL of chloroform and the solvent completely evaporated in a nitrogen atmosphere following 1 h under vacuum. The lipids were then solubilized in 1 mL of 50 mM carboxyfluorescein [2′,7′-bis­(2-carboxyethyl)-5-(and-6)-carboxyfluorescein; Molecular Probes, USA] and the mixture was submitted to ten freeze–thaw cycles (−196 °C/50 °C). The free carboxyfluorescein was separated from the vesicles with trapped carboxyfluorescein by gel filtration in a Superdex 200 10/300 column (Cytiva, USA). LUV leakage promoted by the protein samples was evaluated by the carboxyfluorescein release assay.^34^ The final concentration of LUVs in the experiment corresponds to an absorbance value of 0.1 at 490 nm. All measurements were carried out in a Cary Eclipse fluorescence spectrophotometer (Varian, USA) in which the samples were excited at 490 nm, and the fluorescence was acquired at 518 nm. The absence of leakage (0%) corresponds to the fluorescence of the vesicles at time zero, while 100% leakage was taken as the value of fluorescence intensity obtained after the addition of 1% (v/v) Triton X-100. The degree of LUV permeabilization was calculated by

$$\%leakage=\left[\left({I-I}_{initial}\right)/\left(I_{final}{-I}_{initial}\right)\right]\times 100$$

where *I* is the fluorescence intensity after the addition of the protein, while *I*_initial_ is the initial fluorescence of the LUVs and *I*_final_ is the fluorescence after the addition of 1% Triton X-100. Results were expressed as the mean of three experiments ± the standard deviation.

### Cell Viability

Nondifferentiated SH-SY5Y cell line (Merck, USA) was seeded into 96-well culture plates at density of 1 × 10^5^ cells/mL. The ability of fS194, incubated with or without mWT, to affect the viability of SH-SY5Y cells was determined by using the assay of conversion of 3-(4,5-dimethylthiazol-2-yl)-2,5-diphenyltetrazolium bromide (MTT) to formazan, which is a potential indicator of cell viability.^35^ Monomeric WT at a concentration of 70 μM was incubated with 0, 5, 10, or 20% (% m/m) of fS194 for 20 h. Cells were treated with a final concentration of 20 μM mWT in the presence or absence of fS194. Treated cell cultures were kept at 37 °C in a humidified 5% CO_2_ and 95% air chamber for 24 h. MTT (5 mg/mL) was added to all the wells and allowed to incubate in the dark at 37 °C for 4 h. The reaction was terminated with dimethyl sulfoxide (DMSO). The formation of the MTT formazan product was measured through the absorbance at 540 nm using a Microplate reader. MTT assays were performed in triplicate.

#### Molecular Docking

Molecular docking calculations were carried out using AutoDock Vina,^36^ with two different systems. In the first one, the structure of the receptor, corresponding to the original αSyn monomer (micelle-bound αSyn), was downloaded from the protein data bank (PDB ID 1XQ8).^37^ This structure displays, beginning from the N-terminal, an α helix until residue 36, an unstructured turn until residue 44, a long α helix until residue 92, and an unstructured region from residue 93 until the C-terminal (residue 140). In the second system, we used, as a receptor, equilibrated αSyn monomeric structures obtained as final configurations resulting from 3000 ns MD simulations.^38^ For both systems, the same ligand was used, namely, the structure of the short peptide segment S194, which was obtained from residues 194–203 from the chain A of the SARS-CoV-2 Spike trimer structure PDB ID 6VXX.^39^ The entire receptor structure was inside the searching box (blind docking), and the exhaustiveness parameter was set to 10. The dockings were run in triplicate and yielding, each replica, 9 configurations. In the case of the experimental structure from the protein data bank, the docking scores were rather small (best ranked, binding energy −5.2 kcal/mol), pointing out a moderate interaction, whereas in the case of optimized receptor structures, the best ranked docking poses exhibited strong interactions (binding energy of −7.0 kcal/mol). The docking results were also analyzed with AutoDock Tools^40^ to investigate the pattern of interacting residues.

#### Molecular Dynamics

The best-ranked docked configurations were the starting points for the MD simulations. The structure preparation and simulation setup were done using CHARMM-GUI^41^ choosing the CHARMM36 force field.^42^ The systems were prepared in a simulation box with periodic boundary conditions, solvated using TIP3P water.^43^ Sodium and chloride ions were added, corresponding to the physiological concentration. All simulations were carried out with GROMACS.^44^ The electrostatic interactions were calculated with the Smooth Particle Mesh Ewald method.^45^ The systems were first minimized with the steepest descent algorithm, followed by a 125 ps simulation with position restraints, 1 ns equilibration, and 250 ns production phase in the *NPT* ensemble, with a Parrinello–Rahman barostat^46^ (reference pressure 1 bar) and v-rescale thermostat^47^ (reference temperature 310 K). The generated trajectories were further analyzed with standard GROMACS tools yielding useful metrics such as RMSD, Distances, H-Bonds, Radius of gyration, and solvent accessible surface area to quantify the structural and thermodynamic stability of the complexes and interaction patterns.

## Binding Free-Energy Calculations

The free energy of binding between the ligand (S194) and receptor (αSyn) was calculated using the Molecular Mechanics Poisson–Boltzmann Surface Area (MM/PBSA) method,^48^ with the GROMACS-compatible program gmx_MMPBSA^49^ using its default parameters. In the MM/PBSA method, the free energy of binding is estimated as a sum of bonded and nonbonded (either intramolecular or intermolecular interaction contributions) and a free energy of solvation, which comprises a polar solvation term, calculated solving the Poisson–Boltzmann equation, and a nonpolar solvation term, usually calculated as a function of the solvent-accessible surface area. The program gmx_MMPBSA also allows a decomposition analysis, mapping the most relevant interacting residues along the simulation trajectory. These calculations considered 250 configurations (snapshots) obtained from the molecular dynamics trajectories for each system. In addition to the gmx_MMPBSA free-energy calculations, we estimated the strength of interaction using the approach of Bellaiche and Best.^18^ The free energy is computed as a function of an order parameter: the minimum distance *r*_min_ between αSyn and S194:

$$G\left(r_{\min}\right)=-RT\ln\left(p\left(r_{\min}\right)\right)$$

This estimate can be applied to our systems since the combined simulations are long enough to sample some events of detachment between αSyn and S194. A histogram of the *r*_min_ is built and, based on the free-energy profile G (*r*_min_) × *r*_min_, the cutoff *r*_cut_ distance separating the bound state from unbound state is found (0.5 nm in our systems). The bound probability is defined as the integral of *p*(*r*_min_) from zero to *r*_cu_ and the association constant is defined as the quotient between unbound and bound probabilities. Finally, the free energy of binding is calculated as

$$\Delta_{bind}G^{0}=RT\ln\left[K_{bind}\frac{c_{simul}}{c^{0}}\right]$$

where *c*_simul_ is the molar protein concentration of the systems, which is 1.88 mM in model 1 (29558 water molecules) and 2.01 mM in model 2 (27685 water molecules), respectively.

## Statistics

All experiments were performed as a minimum of three independent repeats. Differences with p-values of less than 0.05 were considered significant (**p <0.01; 0.01 < *p < 0.05; ns: not significant). The results were visualized and analyzed using the OriginPro22 software.

## Supplementary Material



## Notes

Kinetics and TEM images of mS194 incubated with fWT, MD simulation data, proteolytic digestion profile of aggregates obtained using different conditions, and ITC analysis of the interactions of mS194 with fWT versus fA30P (PDF)

## Abbreviations

AD: Alzheimer’s disease
αSyn: α-synuclein
COVID-19: coronavirus disease in 2019
DLS: dynamic light scattering
IPTG: isopropyl-1-thio-β-d-galactopyranoside
ITC: isothermal titration calorimetry
LUV: large unilamellar vesicles
NAC: nonamyloid-β component
NMR: nuclear magnetic resonance
PD: Parkinson’s disease
SARS-CoV-2: severe acute respiratory syndrome coronavirus 2
SEC: size exclusion chromatography
TEM: transmission electron microscopy
ThT: thioflavin-T
WT: wild type.
