# Supplementary material for: An Amyloidogenic Fragment of the Spike Protein from SARS-CoV‑2 Virus Stimulates the Aggregation and Toxicity of Parkinson’s Disease Protein Alpha-Synuclein
Source: ACS Chem Neurosci. 2025 Aug 22;16(17):3385–97. doi: 10.1021/acschemneuro.5c00478 (PMC12412103; doi:10.1021/acschemneuro.5c00478)
Supplement: Supplementary file 1 [file cn5c00478_si_001.pdf]

Supporting information

An amyloidogenic fragment of the Spike protein from SARS-CoV-2 virus stimulates the aggregation and toxicity of the Parkinson´s disease protein alpha-synuclein

João Flavio Gemignani[a], Paulo Augusto Netz[b], Daniel Izecksohn[a], David Dabkiewicz[a], Ming-Hao Li[c], Adalgisa Felippe Wiecikowski[d], David Eliezer[c], Yraima Cordeiro[d], and Cristian Follmer[a*]

[a]Laboratory of Biological Chemistry of Neurodegenerative Disorders, Department of Physical Chemistry, Institute of Chemistry, Federal University of Rio de Janeiro, Rio de Janeiro 21941-909, Brazil. [b]Institute of Chemistry, Federal University of Rio Grande do Sul, Porto Alegre 91501-970, Brazil. [c]Department of Biochemistry, Weill Cornell Medical College, New York, New York 10065. [d]Faculty of Pharmacy, Federal University of Rio de Janeiro, Rio de Janeiro, 21941-902, Brazil.

***Corresponding author:**

Tel: 55 21 3938 7752. E-mail: follmer@iq.ufrj.br

ORCID Cristian Follmer 000-0001-6178-0432

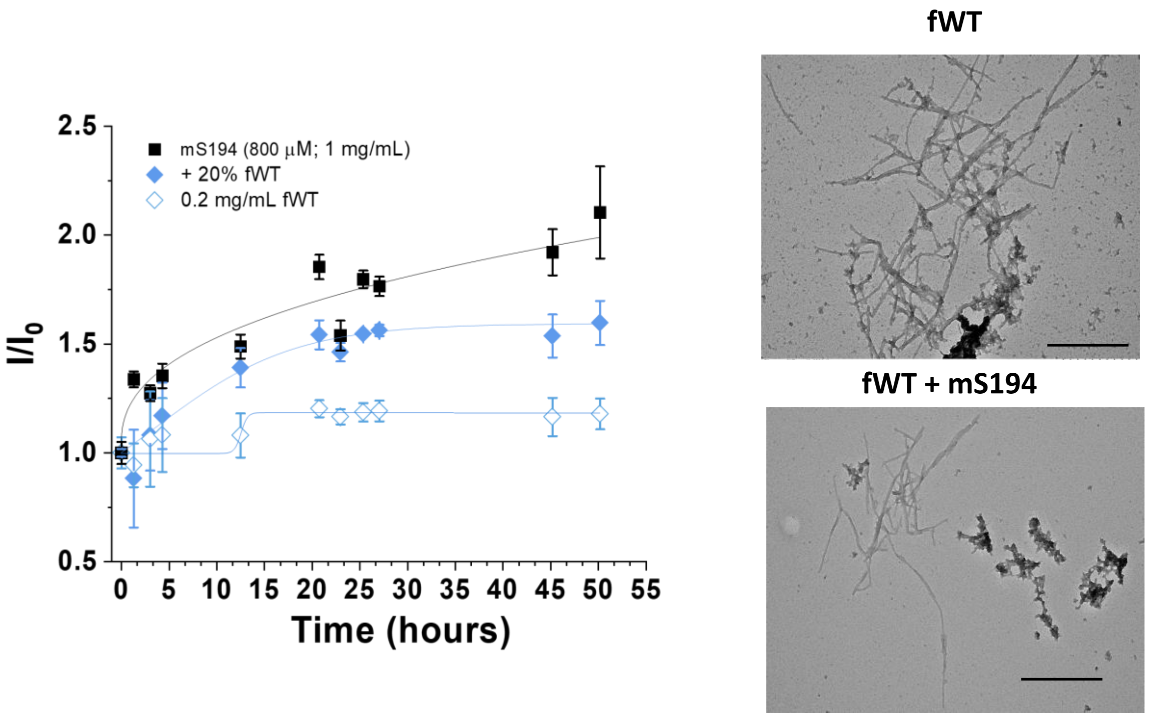

Figure S1. Relative ThT intensity of mS194 (800 μM) incubated with or without 20% of fWT. Results expressed are mean ± standard deviation of six independent assays. Right:TEM images of aggregates at the end of the incubation time (scale bars: 500 nm).

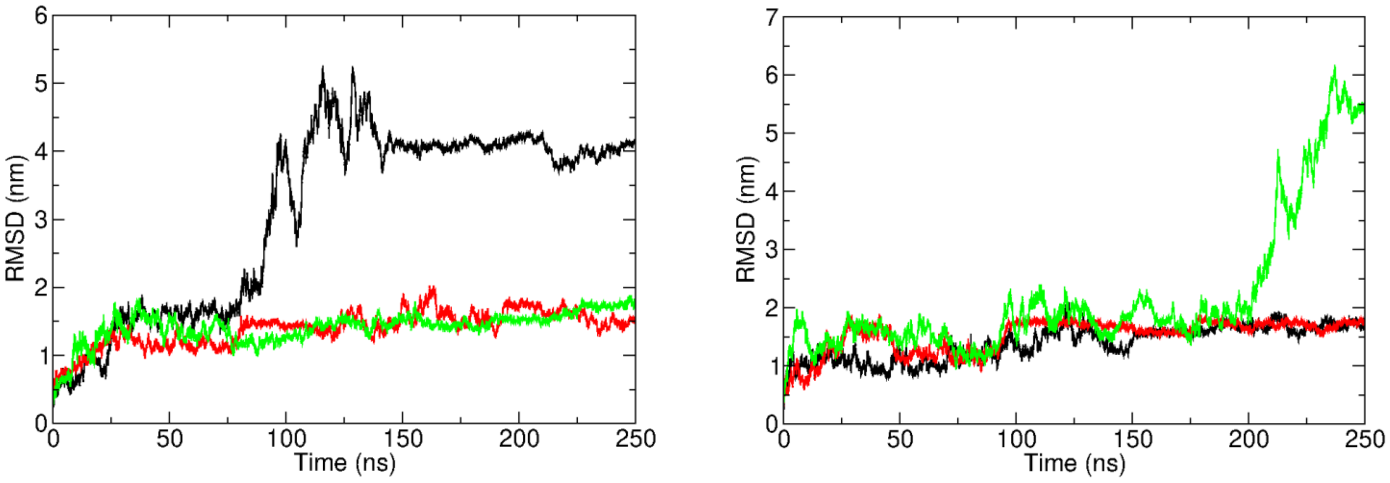

Figure S2. Root mean square deviation (RMSD) considering all atoms of αSyn and S194, for model 1 (left) and model 2 (right). Black: replicate 1, red: replicate 2, green: replicate 3.

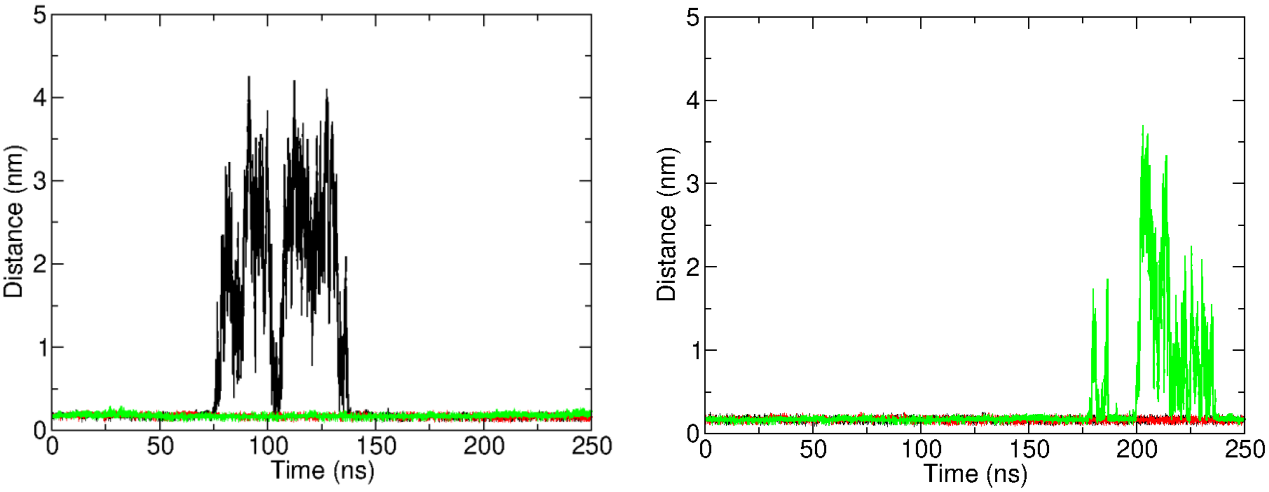

Figure S3. Minimum distance between αSyn and S194, for model 1 (left) and model 2 (right). Black: replicate 1, red: replicate 2, green: replicate 3. The events of unbinding and binding can be seen in model 1, replicate 1 and model 2, replicate 3.

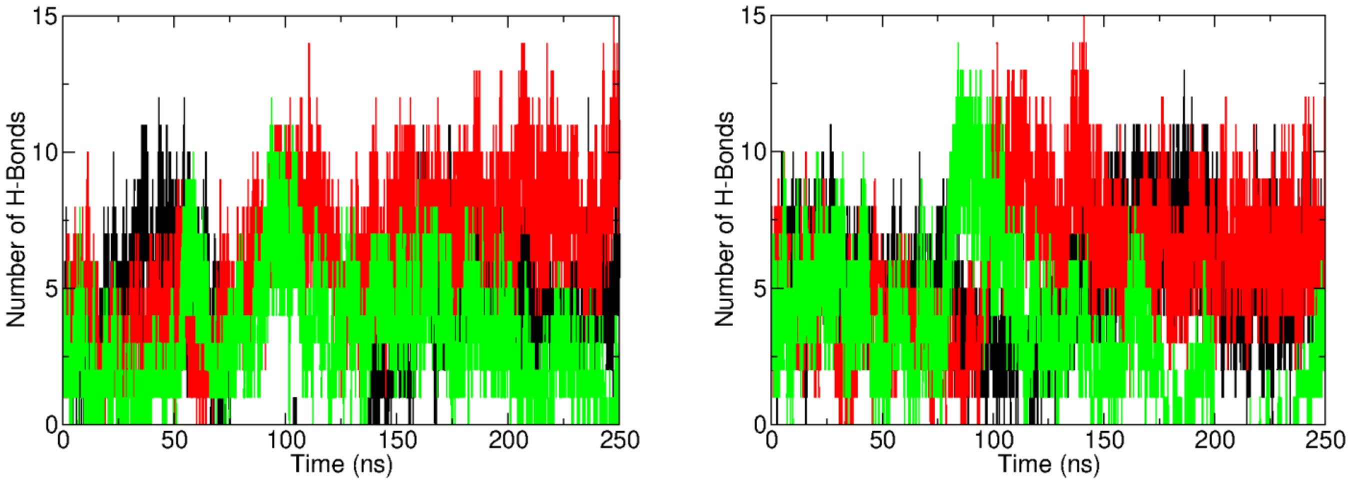

Figure S4. Number of intermolecular hydrogen bonds between αSyn and S194, for model 1 (left) and model 2 (right). Black: replicate 1, red: replicate 2, green: replicate 3.

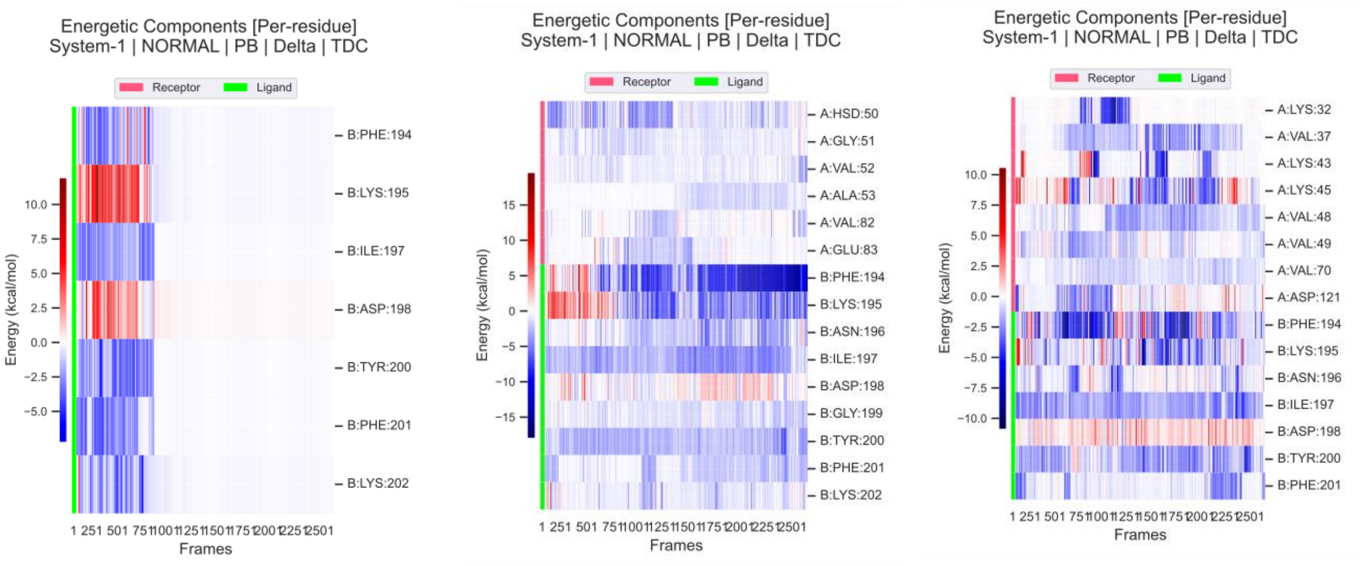

Figure S5. Per-residue contribution (decomposition) analysis, calculated with gmxMMPBSA, for the three replicates of model 1.

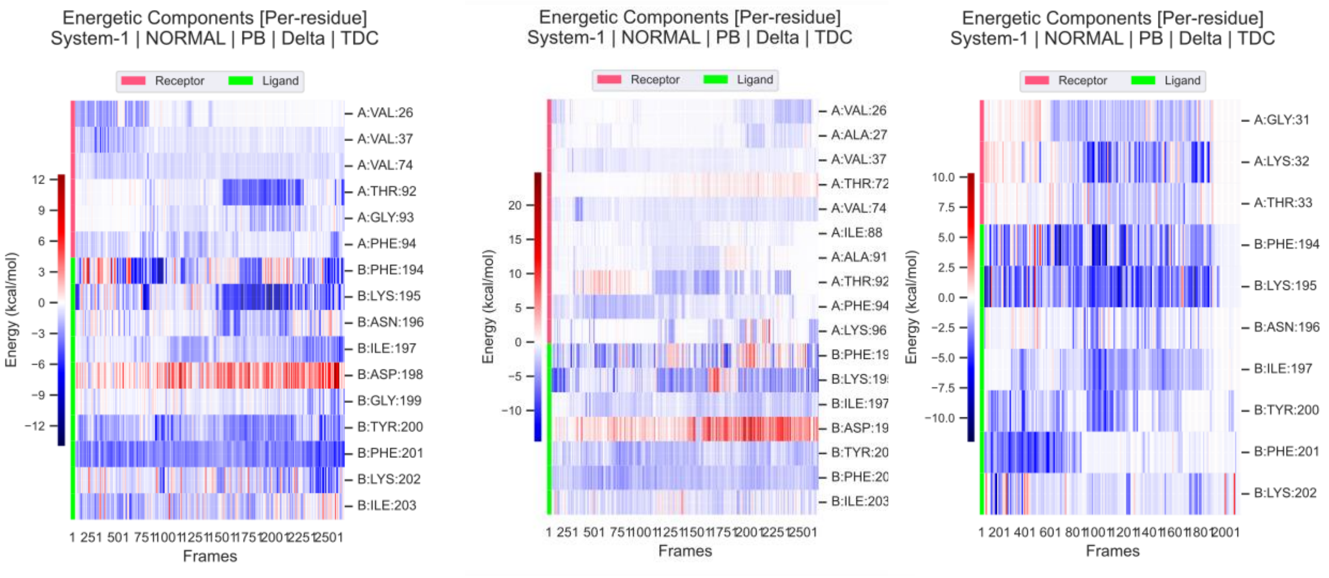

Figure S6. Per-residue contribution (decomposition) analysis, calculated with gmxMMPBSA, for the three replicates of model 2.

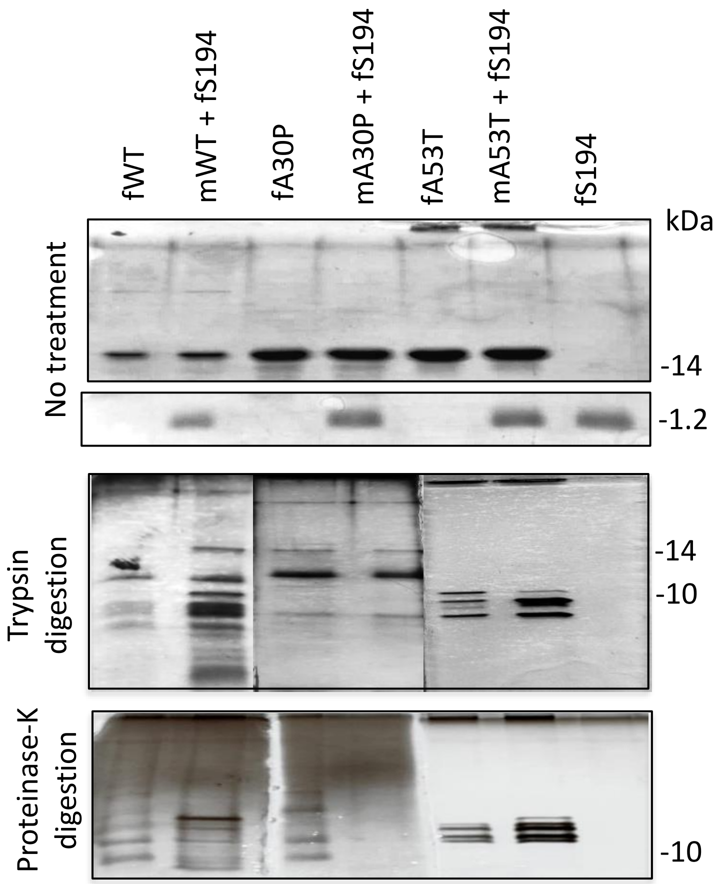

Figure S7. Trypsin or proteinase K proteolytic fragmentation of the samples obtained from the incubation of monomeric αSyn (WT or variants) with or without 5% fS194, in comparison with typical fibrils of αSyn. The gel below the untreated samples is an offset to show mS194 bands (~1 kDa). Position and size (kDa) of molecular weight markers (1.2, 10 and 14 kDa) are indicated on the right side.

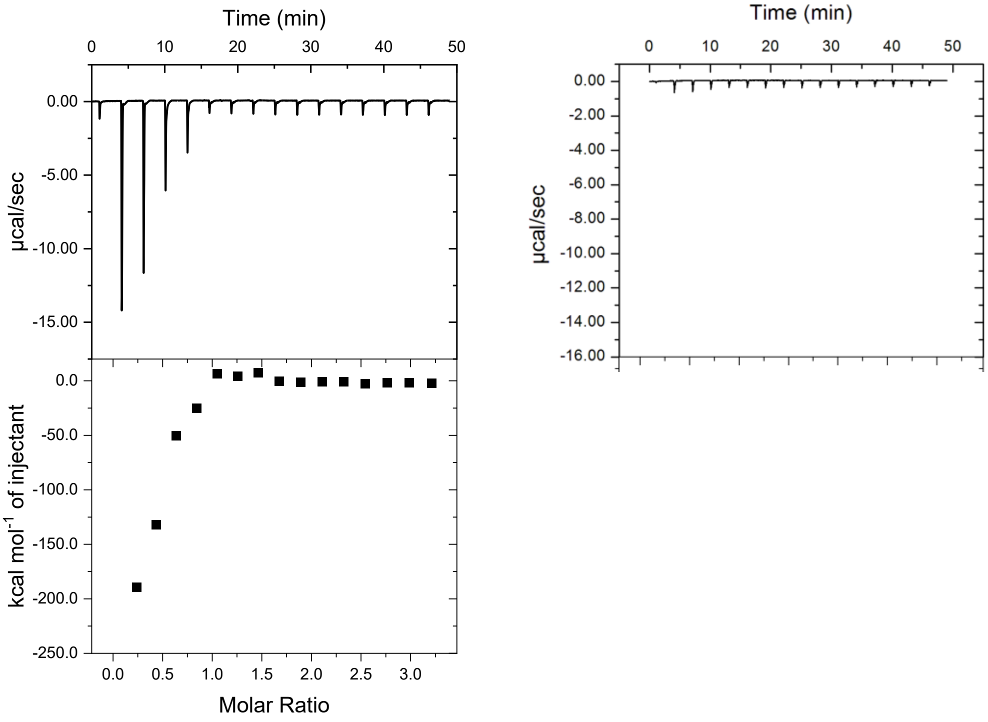

Figure S8. ITC-measured heat release curves (baseline corrected raw data) for fWT and fA30P, respectively, at a concentration of 10 µM (in relation to the monomer) upon titration with 2 µL of 200 µM of mS194 / injection at 25 °C.
